# Self-testing for symptoms and signs of prodromal alpha-synucleinopathies: results from the Tasmanian ISLAND Sleep Study

**DOI:** 10.1007/s11357-025-01757-9

**Published:** 2025-07-01

**Authors:** Samantha Bramich, Alastair J. Noyce, Anna E. King, Eddy Roccati, Sharon L. Naismith, James C. Vickers, Jane Alty

**Affiliations:** 1 Wicking Dementia Research and Education Centre, University of Tasmania, Hobart, TAS Australia; 2 Centre for Preventive Neurology, Wolfson Institute of Population Health, Queen Mary University of London, London, United Kingdom; 3 Brain and Mind Centre, University of Sydney, Sydney, NSW Australia; 4 School of Medicine, University of Tasmania, Hobart, TAS Australia; 5 Neurology Department, Royal Hobart Hospital, Hobart, TAS Australia

**Keywords:** REM sleep behaviour disorder, TAS Test, Neurodegeneration, Sleep disorders, Parkinson’s disease, Dementia

## Abstract

Isolated rapid eye movement (REM) sleep behavior disorder (iRBD) may occur at least 10 years before a clinical diagnosis of an α-synucleinopathy, such as dementia with Lewy bodies (DLB), Parkinson’s disease (PD) or multiple system atrophy (MSA). The extent to which people with iRBD manifest subtle features of these conditions is an area of active research and whether these changes can be measured remotely in the community is an important aim. Better characterisation may aid early detection and monitoring for people at risk of neurodegeneration. The aim of this study was to investigate remote tests of subjective and objective features of α-synuclein-related conditions in probable iRBD (pRBD). We hypothesised that α-synuclein-related symptoms and signs would be more frequent in pRBD than healthy controls. 2,891 participants aged 50 + from the Tasmanian ISLAND Sleep Study completed the REM Sleep Behavior Disorder Single-Question Screen (RBD1Q), and several online questionnaires and unsupervised objective assessments of motor, cognitive and olfactory function. People with pRBD (*n* = 267; mean (SD) age 63 (7.6) years; 53% female) reported more α-synuclein-related features than controls (*n* = 2,624; mean (SD) age 64 (7.7) years; 75% female), especially orthostatic intolerance, neuropathic pain, falls, olfactory impairment and hand motor dysfunction. These results show that people with pRBD exhibit a range of symptoms and signs of α-synuclein-related conditions that can be measured using remote (online and postal) assessment tools. Remote monitoring in the community may aid early detection of progression to DLB, PD and MSA and facilitate enrolment into clinical trials.

## Introduction

Dementia with Lewy bodies (DLB), Parkinson’s disease (PD) and multiple system atrophy (MSA) are some of the fastest-growing neurological conditions in the world [1–3]. They are all forms of ‘α-synucleinopathies’—neurodegenerative diseases characterised by the abnormal deposition and accumulation of α-synuclein in the central and peripheral nervous systems [4]. The earliest features of these α-synuclein-related conditions include several non-motor symptoms pertaining to constipation, hyposmia, orthostatic hypotension, and depression [5], and these can occur many years before the overt classical motor and cognitive signs, such as slowed gait or memory impairment [6].

One of the earliest and most specific symptoms of prodromal α-synucleinopathy is isolated rapid eye movement (REM) sleep behavior disorder (iRBD) [7]. This sleep disorder is characterised by muscle atonia and dream enactment behaviour during REM sleep, often involving limb movements and vocalisations, and can occur at least 10 years before a diagnosis of DLB, PD or MSA [7]. iRBD is thus a key indicator of α-synucleinopathy risk, with recent studies showing that 70–90% of people with iRBD go on to develop either DLB (50% of cases), PD (45% of cases), or MSA (5% of cases) [8].

Phenoconversion of iRBD to DLB, PD or MSA occurs in a gradual manner, with increasing motor and non-motor features developing over several years [9], but our understanding of which α-synucleinopathy (DLB, PD or MSA) is likely to develop, and how quickly, remains unclear. Precision phenotyping in iRBD using accessible home-based tests would allow for regular repeated measures and would improve our understanding of prognostication and transform clinical trial recruitment. It is recognised, for example, that when iRBD co-occurs with certain non-motor symptoms such as olfactory dysfunction, there is a substantially increased risk of progression to DLB and PD [10]. Although olfactory dysfunction can already be measured remotely with standardised tests such as the University of Pennsylvania Smell Identification Test (UPSIT) [11], their cost is high for regular repeated measures. It is necessary to find new ways of detecting and tracking non-motor symptoms in iRBD, alongside olfactory testing.

Similarly, current assessments of motor features in iRBD are generally only accessible in-person, and/or require a clinician or expert assessor to rate the severity of deficits. Usually, part 3 of the Movement Disorders Society revision of the Unified Parkinson's Disease Rating Scale [12] (MDS-UPDRS-3) is used, which requires an expert rater to systematically examine for motor changes in tasks such as finger-tapping and gait. Typically, people with iRBD score in the ‘normal’ range [10, 13], signifying that very few motor changes are detectable using this method. This contrasts with recent evidence that people with iRBD are frequently aware of subtle motor changes [14] and emphasises that further research is needed to develop new tools. Motor function measured with the Alternate Tap Test has been shown to be slower in iRBD (147.4 tap/min) than in age-matched healthy controls (191.3 tap/min) [15], but this is an in-person assessment and there is a pressing need to see how it fares when unsupervised in the community [16]. In preparation for potentially disease modifying clinical trials, and to improve access to diagnostic options in communities without easily accessible specialist services, it is imperative that changes in the severity of symptoms and signs can be measured easily and remotely in a home environment.

In this study, we assessed a range of remote unsupervised tests of α-synuclein-related features in a large community-dwelling cohort (*n* = 2,891) in Australia’s island state of Tasmania. We aimed to evaluate if low-cost remote tests could detect differences in self-identified ‘probable’ RBD (pRBD) compared to controls, as a first step towards developing a suite of online/home-based tests that can be used to assess features of iRBD and α-synucleinopathies, and supplement in-person assessments. We hypothesised that these remote, unsupervised tests would detect more motor and non-motor α-synuclein-related features in those with pRBD compared to controls.

## Methods

### Participants

Participants were recruited from the Island Study Linking Ageing and Neurodegenerative Disease (ISLAND) Project and the general population of Tasmania, Australia [14]. The ISLAND Project is a public health initiative that was launched in 2019 by the Wicking Dementia Research and Education Centre at the University of Tasmania; it aims to build dementia risk management self-efficacy and decrease dementia risk in Tasmanians, and a detailed protocol has been published [17]. The ISLAND Sleep Study is a sub-study of the ISLAND project that aims to detect and characterise iRBD in this Tasmanian community sample; the protocol has been published in Bramich et al. [14]. In brief, methods of recruitment included community poster and leaflet advertisements placed in hospitals, supermarkets and community group buildings, and email invitations. Eligibility criteria for this study were being (i) a resident in Tasmania, (ii) aged 50 years or older, and (iii) a participant in the ISLAND Project. New participants for the ISLAND Sleep Study were required to sign up to the ISLAND Project if they were not already a participant, to allow for data collaboration between the two research projects and collection of detailed demographic data. Participants were excluded from the analysis if they reported a current diagnosis of DLB, PD or MSA (*n* = 18).

This study was approved by the University of Tasmania Human Research Ethics Committee (HREC references 26435, 29005 and 18264) and was carried out according to the National Statement on Ethical Conduct in Human Research (2022). Participants provided consent online as part of the ISLAND Project procedures [17].

### Data collection

Data for the wider ISLAND Project was collected between August 2021 and December 2023, including online questionnaires on demographics, health surveys and mood [17]. ISLAND Sleep Study participants completed a battery of online health and sleep questionnaires between June and August of 2022, that were presented in a fixed order (see [14] and Table 1); they could choose to complete these in one sitting or take as many breaks as they wished. Completing each part of the questionnaire was not compulsory and participants were free to move on to the next question even if there was missing data. The sleep questionnaires included the REM Sleep Behavior Disorder Single-Question Screen (RBD1Q) that asks, “Have you ever been told, or suspected yourself, that you seem to ‘act out your dreams’ while asleep (for example, punching, flailing your arms in the air, making running movements, etc.)?” [18]. It has a reported sensitivity of 80% and a specificity of 75.3% for iRBD in older adult populations, when compared to the gold standard video polysomnography (vPSG) diagnostic criteria, making it one of the most reliable screening measures for RBD currently available [19]. Those that screened positive on the RBD1Q (responded ‘yes’) were defined as pRBD and those that screened negative as controls.

**Table 1:** Online, remote assessments of health and sleep symptoms

Online/Remote Assessment	Test Domain
REM Sleep Behavior Disorder Single-Question Screen (RBD1Q)	Probable iRBD
Baylor Functional Assessment Scale (BFAS)—adapted	Parkinsonism motor symptoms
Composite Autonomic Symptom Score-31(COMPASS-31)	Autonomic function
Pittsburgh Sleep Quality Index (PSQI)	Sleep quality
The Short Form-McGill Pain Questionnaire-2 (SF-MPQ-2)	Pain
The Hospital Anxiety and Depression Scale (HADS)	Anxiety and depression
TAS Test Alternate Tap Test	Hand motor function
Cambridge Neuropsychological Test Automated Battery (CANTAB) + 1 self-report cognitive question	Cognitive function
University of Pennsylvania Smell Identification Tests (UPSIT)	Olfactory function

**Fig. 1:**
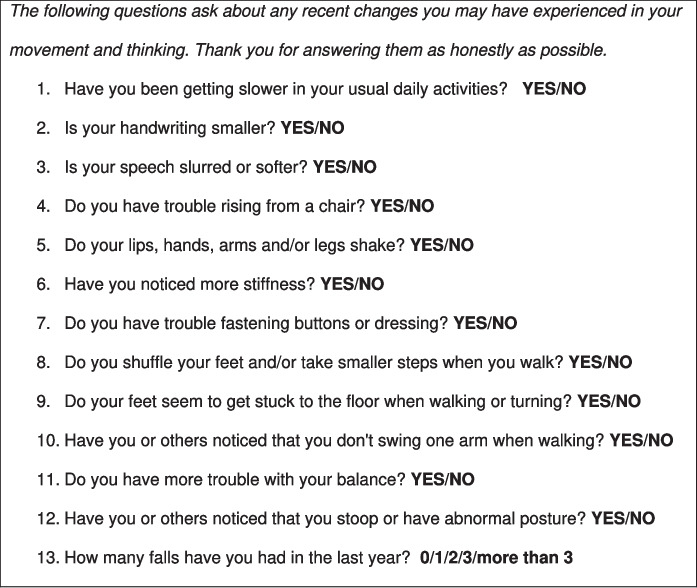
Adapted version of the Baylor Functional Assessment Scale

An adapted version of the Baylor Functional Assessment Scale (BFAS) [20] was used to investigate prodromal parkinsonism symptoms (see Fig. 1). This self-report questionnaire has been validated to differentiate PD from controls [20], and was designed to assess changes in movement, from the participant’s perspective. To the twelve published questions (which were scored yes = 1, no = 0; see Fig. 1), one additional question was added in this study to assess for falls, which are relevant to α-synucleinopathies: “How many falls have you had in the last year?” (0/1/2/3/more than 3).

The Composite Autonomic Symptom Score-31 (COMPASS-31; [21]) scale was used to assess for subjective autonomic function changes, and these were categorised into six domains: orthostatic intolerance and vasomotor, secretomotor, gastrointestinal, bladder, and pupillomotor dysfunction. It has good correlation with objective autonomic test results in people with PD, particularly for orthostatic hypotension symptoms [22]. The Pittsburgh Sleep Quality Index (PSQI [23]), was used to assess overall sleep quality, along with seven individual components of subjective sleep quality, sleep latency, sleep duration, sleep efficiency, sleep disturbances, use of sleep medications, and daytime dysfunction due to sleepiness. It has been validated for the detection of poor sleep quality in people with DLB and PD [24, 25] and is regularly used to assess sleep quality in people with α-synucleinopathies [26–28]. The Short Form-McGill Pain Questionnaire-2 (SF-MPQ-2; [29]) was used to assess major symptoms of both neuropathic and non-neuropathic pain, and consisted of twenty-two sub-qualities of pain, which are further divided into four main categories of pain: *continuous pain descriptors* (6 items): “throbbing pain”, “cramping pain”, “gnawing pain”, “aching pain”, “heavy pain”, and “tender”; *intermittent pain descriptors* (6 items): “shooting pain”, “stabbing pain”, “sharp pain”, “splitting pain,” “electric-shock pain”, and “piercing”; *predominantly neuropathic pain descriptors* (6 items): “hot-burning pain”, “cold-freezing pain”, “pain caused by light touch”, “itching”, “tingling or ‘pins and needles,’”, and “numbness”; and *affective descriptors* (4 items): “tiring-exhausting”, “sickening”, “fearful”, and “punishing-cruel”. The scale has previously been objectively validated in patients with chronic pain using double-blind, randomised clinical trials of pain relief medication [30] and against several other pain scales in people with PD [31]. The Hospital Anxiety and Depression Scale (HADS) [32] was extracted from the ISLAND Project (completed in June 2022) to assess for the presence of depressive and anxiety-related symptoms, and is a well validated measure in older adults [33] as well as in people with α-synucleinopathies [34, 35].

To measure objective motor features, TAS Test was administered online in August 2021 [36]. This is an unsupervised online suite of motor, cognitive and speech-based tasks that has been developed at the University of Tasmania and uses a standard laptop or desktop computer; the detailed protocol [36], and several validation papers have been published [37–39]. The TAS Test Alternate Tap Test is a computerised assessment of hand motor function based on the Bradykinesia Akinesia Incoordination (BRAIN) tap test [40] whereby participants tap the “S” and “;” keys on the computer keyboard alternately with their dominant hand as fast as possible for 30s and then repeat this with their non-dominant hand. Several component measures can be extracted from this such as key tapping frequency, variability and pauses [36, 40, 41], and the alternate key tapping task has been validated in differentiating PD patients and those with iRBD from healthy controls, and against clinical rating scales in those with PD [40, 42–44].

In addition, cognitive function was assessed using self-report and objective measures. Participants were asked “Have you noticed persistent symptoms or difficulties with your thinking/memory/concentration for at least 3 months?” (scored yes = 1, no = 0), in addition to completing the paired associates learning (PAL) and spatial working memory (SWM) tasks of the Cambridge Neuropsychological Test Automated Battery (CANTAB; [45]). The CANTAB assessments were administered online in October 2021 as part of the annual ISLAND project data collection [17], whilst the self-report question was administered between June and August 2022. The CANTAB has been validated in identifying early cognitive changes in neurodegenerative disorders such as Alzheimer’s disease and PD [45]. The PAL task specifically assesses visual episodic memory through learning and recall of colourful abstract patterns presented within 6 possible locations on a computer screen. The number of errors made on the PAL 6 shapes trial was selected for analysis because this is highly sensitive to mild cognitive impairment (MCI) [46–48]. The SWM task assesses the ability to retain and manipulate visuospatial information; it involves the presentation of several coloured squares on a computer screen with the aim to locate one token in the array of squares, requiring recall from previous trials [49]. It is sensitive to MCI, specifically of deficits in visual working memory and executive function [47, 50], and has been used to detect cognitive changes in α-synucleinopathies [51].

Olfactory dysfunction was evaluated using UPSIT kits in a sub-sample of participants from the pRBD and control groups. Kits were posted out and returned via pre-paid envelopes between February and December 2023. The UPSIT is a 40-item self-administered ‘scratch and sniff’ test that requires participants to select the correct odour from four options. It is well validated for research and age- and sex-specific normative data have been published [11]. Due to supply constraints, participants completed either the UK or USA version of the kit (33 odours are common to both kits and 7 odours are country-specific).

Additional demographic data was extracted from the ISLAND Project, including age (years), sex (male/female/other/prefer not to say), marital status (single, married, de facto, separated or divorced, widowed), number of children, level of education (primary school, high school, certificate or apprenticeship, diploma/associates degree, bachelor degree, higher university degree, other), employed (yes/no), retired (yes/no), remoteness area (inner regional Australia, outer regional Australia, remote Australia, very remote Australia; [52]), country of birth and Aboriginal/Torres Strait Islander (indigenous Australian) origin (no/yes – Aboriginal/yes—both Aboriginal and Torres Strait Islander/yes—Torres Strait Islander)*.*

### Data analysis

All data cleaning, statistical analyses and figure generation were performed using Rstudio [53]. Numerical data were expressed as mean and standard deviations, and categorical variables as number and percentage. Comparisons between pRBD status and all assessment variables were analysed using either *t*-tests or Chi-squared tests for overall group differences. General linear or logistic models were used to determine significance between the groups. Model 1 was unadjusted, and Model 2 adjusted for age, sex and Aboriginal/Torres Strait Islander origin to exclude any effect of age, sex and indigenous ancestry on outcomes. UPSIT-type (UK and USA) was included as a confounder in the olfactory analyses. For analysis of BFAS variables, survey responses were analysed separately and then scored and summed. All p-values were corrected for multiple comparisons using the Bonferroni correction algorithm, which adjusts the statistical significance level based on the number of tests performed for each analyses [54].

## Results

### Description of pRBD and control cohorts

2,891 (mean (SD) age 64 (7.7) years; 74% female) participants were recruited to this study and 267 (9.2%) screened positive (answered ‘yes’) for pRBD (mean (SD) age 63 (7.6) years; 53% female) with 2,624 screening negative (age 64 (7.7) years; 73% female). Age was positively associated with pRBD (*p* < 0.001), and 4% of participants with pRBD reported being of indigenous Australian (Aboriginal/Torres Strait Islander) origin, compared to 1% of controls (*p* < 0.001) (see Table 2).

**Table 2:** Demographic characteristics of the cohort

	Control (*N* = 2624)	pRBD (*N* = 267)	**Overall** **(*N* = 2891)**
**Age (in years)**			
Mean (SD)	63.9 (7.72)	62.6 (7.59)	63.8 (7.72)
Median [Min, Max]	64.0 [50.0, 91.0]	62.0 [50.0, 88.0]	63.0 [50.0, 91.0]
**Gender**			
Female	1987 (75.7%)	147 (55.1%)	2134 (73.8%)
Male	631 (24.0%)	119 (44.6%)	750 (25.9%)
Other	4 (0.2%)	1 (0.4%)	5 (0.2%)
Prefer not to say	2 (0.1%)	0 (0%)	2 (0.1%)
**Marital Status**			
Married	1533 (58.4%)	174 (65.2%)	1707 (59.0%)
Separated or divorced	353 (13.5%)	32 (12.0%)	385 (13.3%)
De-facto	274 (10.4%)	25 (9.4%)	299 (10.3%)
Single	206 (7.9%)	19 (7.1%)	225 (7.8%)
Widowed	171 (6.5%)	10 (3.7%)	181 (6.3%)
Other	15 (0.6%)	4 (1.5%)	19 (0.7%)
Prefer not to say	4 (0.2%)	0 (0%)	4 (0.1%)
Missing	68 (2.6%)	3 (1.1%)	71 (2.5%)
**Highest level of education**			
Higher University degree (Honours, Graduate Diploma, Masters or PhD)	847 (32.3%)	94 (35.2%)	941 (32.5%)
Bachelor's Degree	573 (21.8%)	43 (16.1%)	616 (21.3%)
Diploma/Associate Degree	478 (18.2%)	50 (18.7%)	528 (18.3%)
High School	332 (12.7%)	37 (13.9%)	369 (12.8%)
Certificate or Apprenticeship	258 (9.8%)	37 (13.9%)	295 (10.2%)
Other	84 (3.2%)	5 (1.9%)	89 (3.1%)
Primary School	1 (0.0%)	0 (0%)	1 (0.0%)
Missing	51 (1.9%)	1 (0.4%)	52 (1.8%)
**Currently employed**			
No	1488 (56.7%)	145 (54.3%)	1633 (56.5%)
Yes	1090 (41.5%)	121 (45.3%)	1211 (41.9%)
Missing	46 (1.8%)	1 (0.4%)	47 (1.6%)
**Retired**			
No	210 (8.0%)	29 (10.9%)	239 (8.3%)
Yes	1312 (50.0%)	128 (47.9%)	1440 (49.8%)
N/A	0 (0%)	1 (0.4%)	1 (0.0%)
Missing	1102 (42.0%)	109 (40.8%)	1211 (41.9%)
**Number of children**			
Mean (SD)	2.11 (2.44)	1.96 (1.41)	2.10 (2.37)
Median [Min, Max]	2.00 [0, 44.0]	2.00 [0, 11.0]	2.00 [0, 44.0]
Missing	102 (3.9%)	6 (2.2%)	108 (3.7%)
Remoteness Area*			
Inner Regional Australia	1930 (73.6%)	196 (73.4%)	2126 (73.5%)
Outer Regional Australia	664 (25.3%)	67 (25.1%)	731 (25.3%)
Remote Australia	5 (0.2%)	2 (0.7%)	7 (0.2%)
Very Remote Australia	7 (0.3%)	0 (0%)	7 (0.2%)
Missing	18 (0.7%)	2 (0.7%)	20 (0.7%)
**Country of birth**			
Australia	1628 (62.0%)	156 (58.4%)	1784 (61.7%)
UK	318 (12.1%)	24 (9.0%)	342 (11.8%)
New Zealand	56 (2.1%)	3 (1.1%)	59 (2.0%)
Netherlands	22 (0.8%)	2 (0.7%)	24 (0.8%)
Germany	21 (0.8%)	2 (0.7%)	23 (0.8%)
Ireland	12 (0.5%)	1 (0.4%)	13 (0.4%)
Philippines	1 (0.0%)	1 (0.4%)	2 (0.1%)
Poland	3 (0.1%)	0 (0%)	3 (0.1%)
Other	137 (5.2%)	24 (8.6%)	160 (5.5%)
Missing	426 (16.2%)	55 (20.6%)	481 (16.6%)
**Aboriginal and/or Torres Strait Islander origin**			
No	2156 (82.2%)	200 (74.9%)	2356 (81.5%)
Yes, Aboriginal	25 (1.0%)	10 (3.7%)	35 (1.2%)
Yes, both Aboriginal and Torres Strait Islander	2 (0.1%)	0 (0%)	2 (0.1%)
Yes, Torres Strait Islander	1 (0.0%)	0 (0%)	1 (0.0%)
Missing	440 (16.8%)	57 (21.3%)	497 (17.2%)

*NB: Relative geographic remoteness in Australia is measured by calculating road distance from various populated locations [52]

### Motor symptoms

**Fig. 2:**
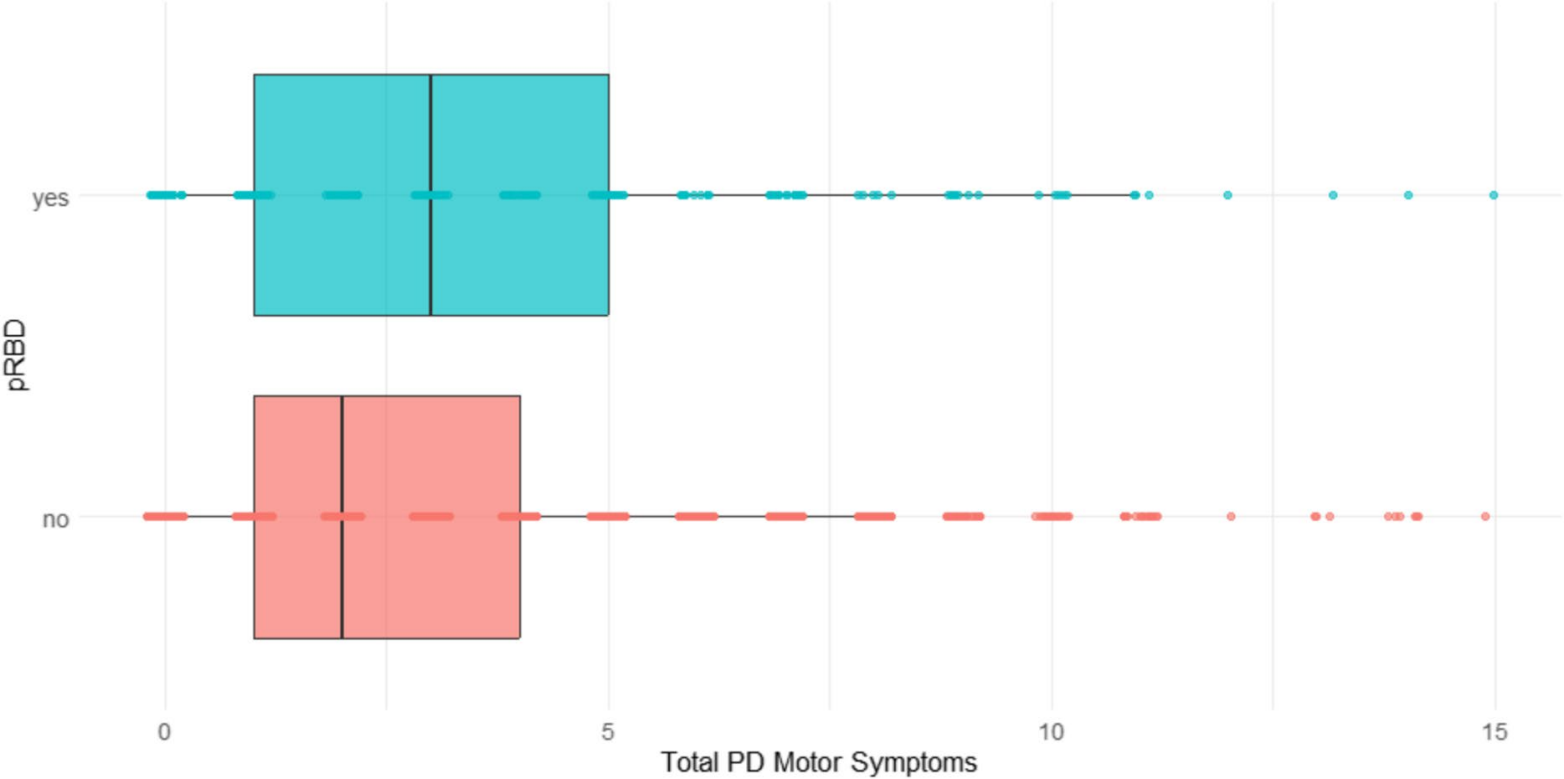
Boxplot of total reported motor symptoms between pRBD and control groups

**Fig. 3:**
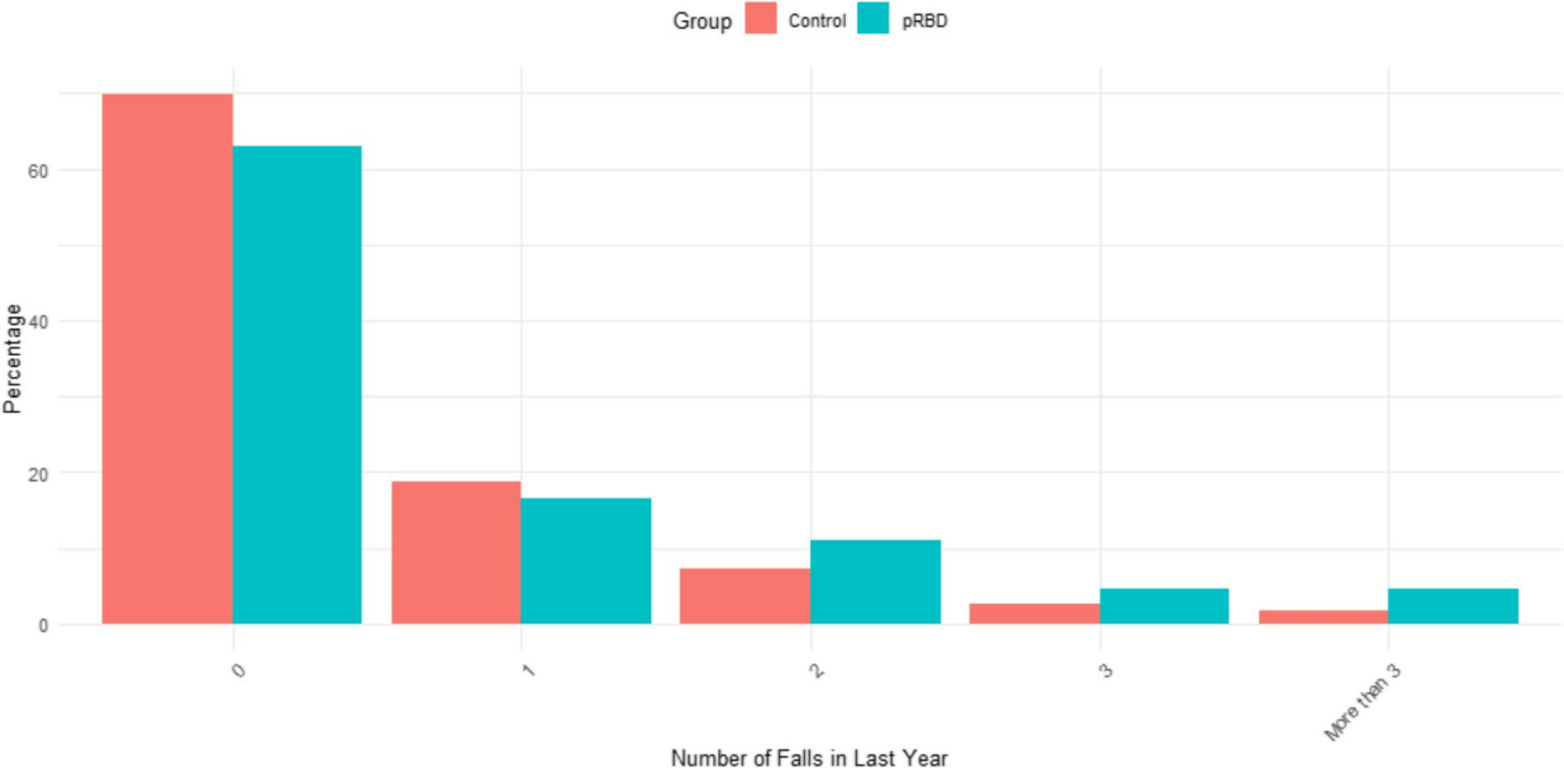
Number of falls reported by those with pRBD and controls within the past three months

2,846 participants (98% of the cohort) completed the adapted version of the BFAS. Group differences between pRBD and controls can be seen in Table 3. In the unadjusted model, those with pRBD reported more total motor symptoms compared to controls (*F* = 24.525, *p* < 0.001), which remained significant after adjusting for age, sex, and Aboriginal and/or Torres Strait Islander origin (*F* = 21.0874, *p* < 0.001; see Fig. 2). Significant differences were also found for several individual BFAS questions after adjusting for confounders, independent of total PD symptom score; the pRBD group reported their speech was more slurred or softer (*z* = 3.21; *p* = 0.001); they had more shaking in their lips, hands, arms and/or legs (*z* = 3.57; *p* < 0.001); difficulty with their feet getting stuck to the floor when walking or turning (*z* = 3.62; *p* < 0.001); more trouble with balance (*z* = 3.84; *p* < 0.001), and more falls in the last year (*z* = 3.62; *p* < 0.001; see Fig. 3).

**Table 3:** Description of reported Parkinsonism symptoms in the cohort (Model 1)*: group scores on the BFAS (adapted) between pRBD and control groups

	Control (*N* = 2581)	pRBD (*N* = 265)	*P*-value
**Have you been getting slower in your usual daily activities?**			
No	1480 (57.3%)	135 (50.9%)	0.112
Yes	1090 (42.2%)	128 (48.3%)	
Missing	11 (0.4%)	2 (0.8%)	
**Is your handwriting smaller?**			
No	2426 (94.0%)	237 (89.4%)	0.010
Yes	143 (5.5%)	27 (10.2%)	
Missing	12 (0.5%)	1 (0.4%)	
**Is your speech slurred or softer?**			
No	2439 (94.5%)	230 (86.8%)	** < 0.001**
Yes	120 (4.6%)	30 (11.3%)	
Missing	22 (0.9%)	5 (1.9%)	
**Do you have trouble rising from a chair?**			
No	2089 (80.9%)	199 (75.1%)	0.073
Yes	468 (18.1%)	63 (23.8%)	
Missing	24 (0.9%)	3 (1.1%)	
**Do your lips, hands, arms and/or legs shake?**			
No	2399 (92.9%)	223 (84.2%)	** < 0.001**
Yes	153 (5.9%)	37 (14.0%)	
Missing	29 (1.1%)	5 (1.9%)	
**Have you noticed more stiffness?**			
No	989 (38.3%)	90 (34.0%)	0.343
Yes	1567 (60.7%)	173 (65.3%)	
Missing	25 (1.0%)	2 (0.8%)	
**Do you have trouble fastening buttons or dressing?**			
No	2358 (91.4%)	228 (86.0%)	0.014
Yes	199 (7.7%)	34 (12.8%)	
Missing	24 (0.9%)	3 (1.1%)	
**Do you shuffle your feet and/or take smaller steps when you walk?**			
No	2351 (91.1%)	240 (90.6%)	0.213
Yes	206 (8.0%)	25 (9.4%)	
Missing	24 (0.9%)	0 (0%)	
**Do your feet seem to get stuck to the floor when walking or turning?**			
No	2462 (95.4%)	247 (93.2%)	0.099
Yes	82 (3.2%)	15 (5.7%)	
Missing	37 (1.4%)	3 (1.1%)	
**Have you or others noticed that you don't swing one arm when walking?**			
No	2527 (97.9%)	260 (98.1%)	0.837
Yes	39 (1.5%)	3 (1.1%)	
Missing	15 (0.6%)	2 (0.8%)	
**Do you have more trouble with your balance?**			
No	1893 (73.3%)	171 (64.5%)	0.002
Yes	675 (26.2%)	90 (34.0%)	
Missing	13 (0.5%)	4 (1.5%)	
**Have you or others noticed that you stoop or have abnormal posture?**			
No	2235 (86.6%)	221 (83.4%)	0.352
Yes	321 (12.4%)	41 (15.5%)	
Missing	25 (1.0%)	3 (1.1%)	
**How many falls have you had in the last year? 0/1/2/3/more than 3**			
0	1789 (69.3%)	166 (62.6%)	** < 0.001**
1	489 (18.9%)	45 (17.0%)	
2	185 (7.2%)	28 (10.6%)	
3	68 (2.6%)	11 (4.2%)	
More than 3	43 (1.7%)	11 (4.2%)	
Missing	7 (0.3%)	4 (1.5%)	

*All scored out of 1, where 1 is ‘yes’ and 0 is ‘no’, apart from frequency of falls.

### Autonomic dysfunction

**Fig. 4:**
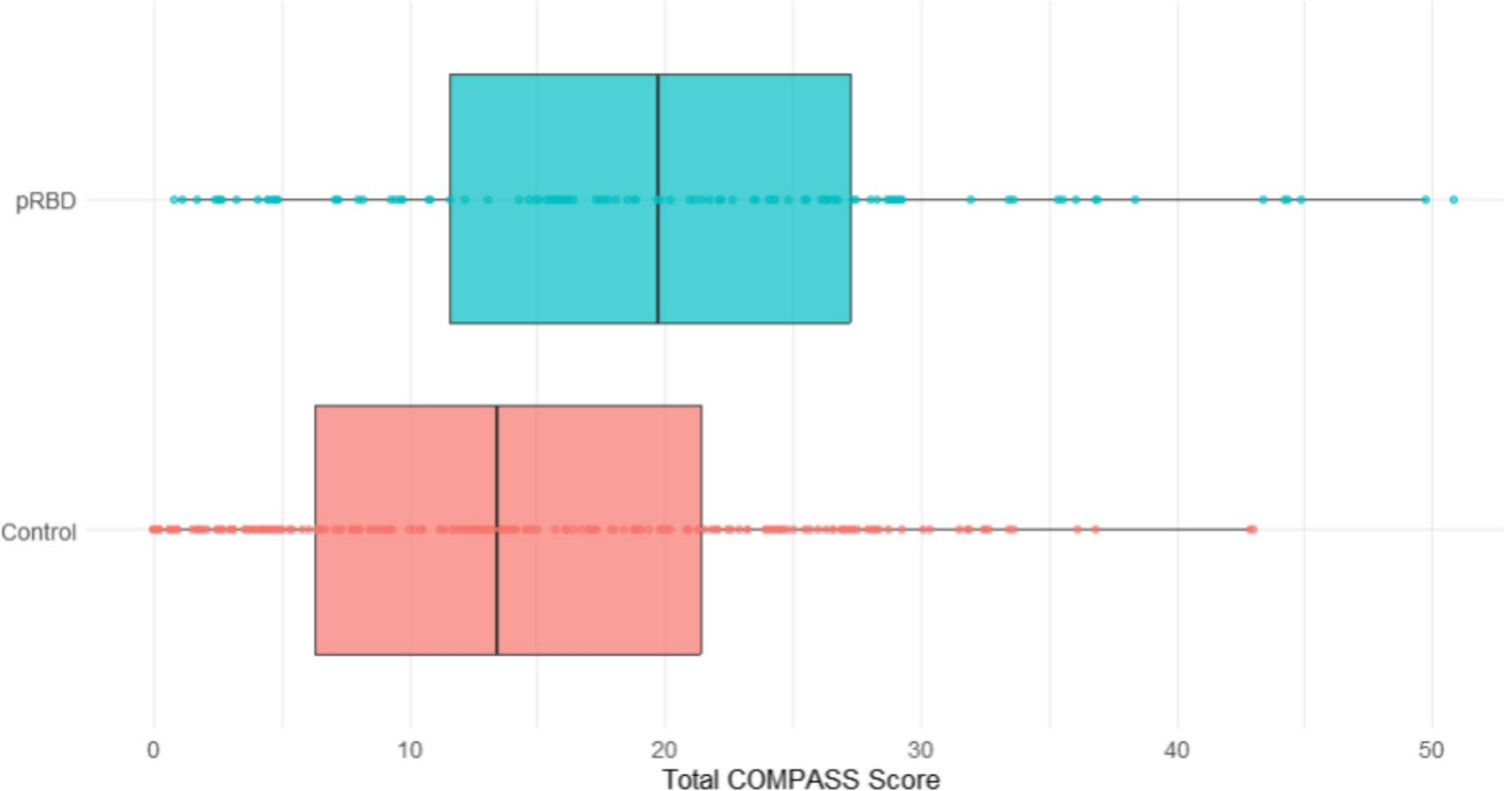
Boxplot of total COMPASS-31 (autonomic) scores between pRBD and control groups

**Fig. 5:**
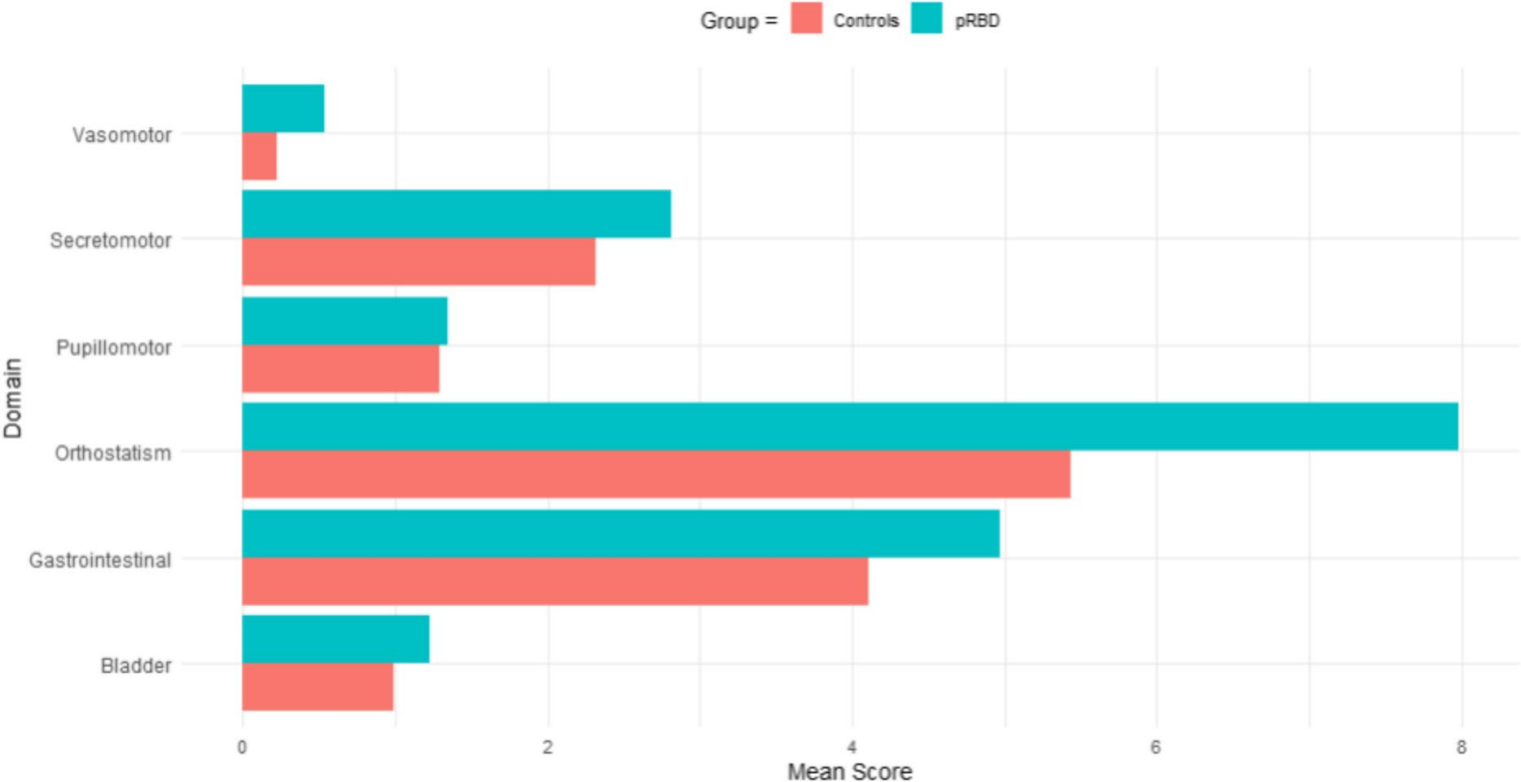
Column chart of COMPASS-31 (autonomic) domain scores between pRBD and control groups

2,586 participants (89% of the cohort) completed the COMPASS-31. In the unadjusted model, those with pRBD reported more autonomic dysfunction symptoms than controls (*F* = 43.126, *p* < 0.001), which remained significant after adjusting for confounders (*F* = 37.433, *p* < 0.001; see Fig. 4). Significant differences were also found for several autonomic function domains after adjusting for confounders, independent of total autonomic function score. People with pRBD reported more orthostatic intolerance (*F* = 29.756; *p* < 0.001), vasomotor dysfunction (*F* = 28.133; *p* < 0.001), gastrointestinal dysfunction (*F* = 12.459; *p* < 0.001), and secretomotor dysfunction (*F* = 6.743; *p* < 0.01). No significant differences were found between the groups for symptoms of bladder or pupillomotor dysfunction (see Fig. 5).

### Pain symptoms

**Fig. 6:**
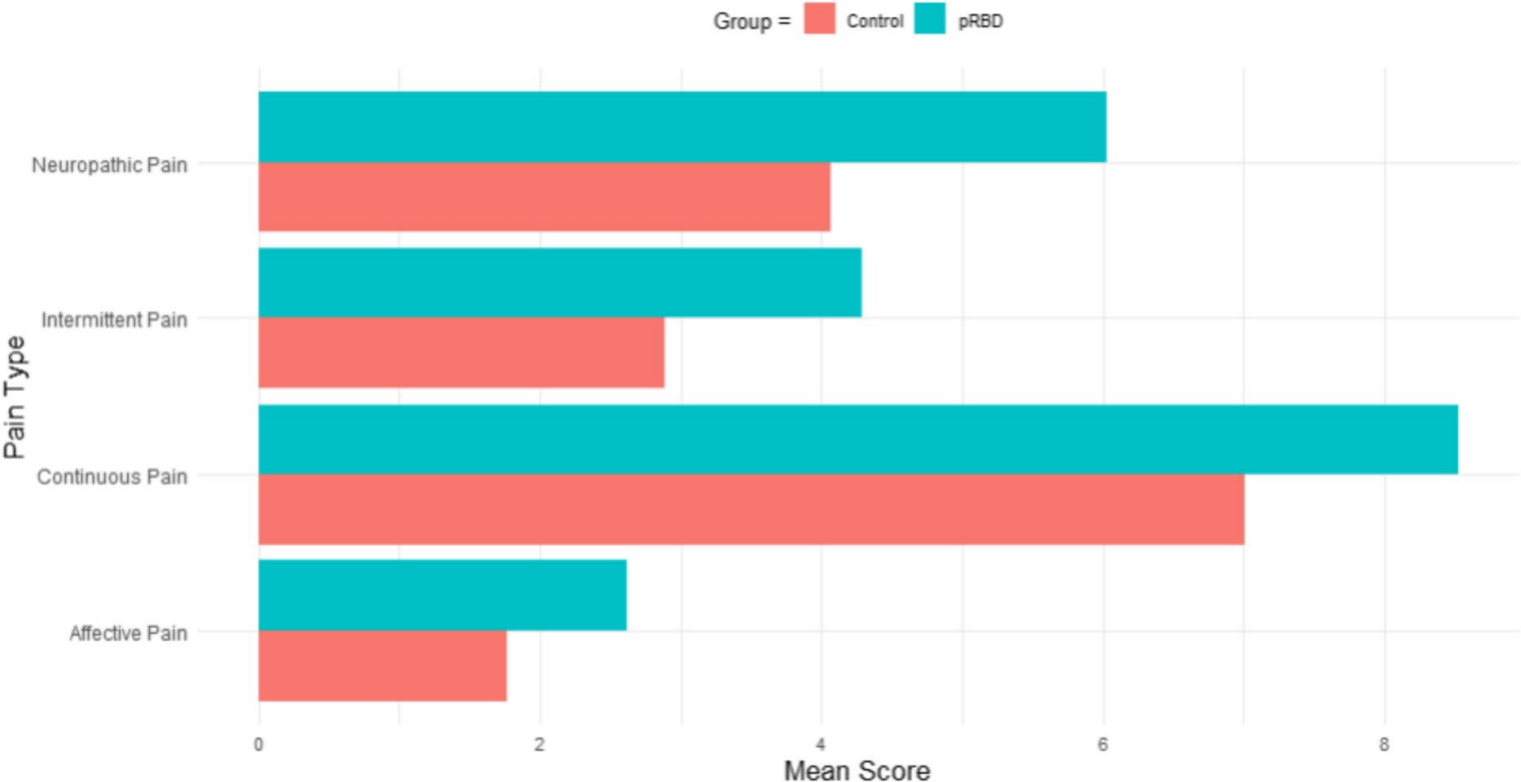
Column chart of pain category scores between pRBD and control groups

2,586 participants (89% of the cohort) completed the SF-MPI-2. In the adjusted analyses, significant group differences were found for all pain categories; in particular, people with pRBD reported more intense neuropathic pain (*F* = 19.927; *p* < 0.001; see Fig. 6) and intermittent pain (*F* = 11.432; *P* < 0.001) than controls. Significant differences were also found between groups for continuous pain (*F* = 8.107; *p* = 0.004), and affective pain (*F* = 8.061; *p* = 0.005) intensity.

### Sleep quality

2,586 participants (89% of the cohort) completed the PSQI. Overall sleep quality was worse in the pRBD compared to the control group (*F* = 7.014; *p* < 0.01); however, this was rendered non-significant after accounting for confounding variables (*F* = 4.383; *p* = 0.18). Significant differences were found in the sub-categories of sleep disturbance (*F* = 28.522; *p* < 0.001) and daytime dysfunction due to sleepiness (*F* = 24.268; *p* < 0.001), with the pRBD group reporting greater symptoms than controls (see Table 4).

**Table 4:** Mean score differences between pRBD and control groups on the Pittsburgh Sleep Quality Index items

	Control (*N* = 2351)	pRBD (*N* = 235)	pRBD (*N* = 235)	*P*-value
**Sleep Quality**				
Mean (SD)	1.15 (0.729)	1.24 (0.760)		0.0623
**Sleep Latency**				
Mean (SD)	1.23 (0.961)	1.26 (0.956)		0.627
**Sleep Duration**				
Mean (SD)	0.56 (0.813)	0.63 (0.845)		0.261
**Sleep Efficiency**				
Mean (SD)	1.04 (1.05)	1.02 (1.07)		0.843
**Sleep Disturbance**				
Mean (SD)	1.56 (0.584)	1.76 (0.596)		** < 0.001**
**Sleep Medications**				
Mean (SD)	0.509 (0.968)	0.562 (1.05)		0.464
**Daytime Dysfunction due to Sleepiness**				
Mean (SD)	0.802 (0.661)	1.06 (0.745)		** < 0.001**
**Total PSQI Score**				
Mean (SD)	6.85 (3.78)	7.54 (3.94)		0.011

### Mood

2,554 participants (88% of the cohort) completed the HADS and people with pRBD reported higher scores for both anxiety (Mean (SD): 6.55 (3.96) versus 5.35 (3.55); *F* = 24.745; *p* < 0.001) and depression (Mean (SD): 3.88 (3.11) versus 3.10 (2.81); *F* = 21.881; *p* < 0.001), after adjusting for confounders. Age and sex were positively associated with pRBD in the adjusted model for anxiety only (*p* < 0.001).

### Hand motor function

1,272 participants (44% of the cohort) completed the online TAS Test Alternate Tap Test. Group differences are shown in Table 5. After adjusting for confounders, the non-dominant hand median time between taps (*F* = 21.445; *p* < 0.001) and key press frequency (*F* = 12.837; *p* < 0.001) was more impaired in people with pRBD compared to controls. No other measures were significantly different between the groups.

**Table 5:** TAS Test Alternate Tap Test (hand motor function) group differences between pRBD and control groups for dominant and non-dominant hand

	Controls (*N* = 1160)	pRBD (*N* = 112)	*P*-value*
***Dominant Hand***			
**Median Time Between Taps (msec)**			
Mean (SD)	481 (86.9)	511 (124)	0.013
Median [Min, Max]	472 [32.0, 867]	502 [32.0, 929]	
**Variability/Rhythm (log)**			
Mean (SD)	0.183 (0.286)	0.184 (0.264)	0.97
Median [Min, Max]	0.0847 [0.0233, 3.44]	0.0998 [0.0191, 1.24]	
**Key Press Frequency (msec)**			
Mean (SD)	21.4 (3.98)	20.3 (4.56)	0.020
Median [Min, Max]	21.0 [4.00, 61.0]	20.0 [3.00, 33.0]	
**Dwell Time on Each Key (msec)**			
Mean (SD)	130 (775)	207 (1210)	0.507
Median [Min, Max]	85.0 [41.0, 17400]	82.0 [50.0, 12900]	
***Non-dominant Hand***			
**Median Time Between Taps (msec)**			
Mean (SD)	510 (93.1)	548 (120)	**0.001**
Median [Min, Max]	500 [38.0, 923]	536 [39.0, 1020]	
**Variability/Rhythm (log)**			
Mean (SD)	0.188 (0.289)	0.193 (0.295)	0.885
Median [Min, Max]	0.0828 [0.0254, 2.13]	0.0917 [0.0155, 1.43]	
**Key Press Frequency (msec)**			
Mean (SD)	20.4 (3.83)	19.0 (3.80)	** < 0.001**
Median [Min, Max]	20.0 [4.00, 61.0]	19.0 [7.00, 28.0]	
**Dwell Time on Each Key (msec)**			
Mean (SD)	129 (587)	251 (1350)	0.345
Median [Min, Max]	101 [40.0, 15300]	101 [55.0, 14200]	

*Bold p-value denotes significant interaction after adjusting for confounding variables

### Cognitive function

2,846 participants (98% of the cohort) completed the self-report cognitive question. After adjusting for confounders, a small significant difference was found between groups, with pRBD reporting more persistent symptoms or difficulties with their thinking/memory/concentration for at least 3 months (*z* = 2.533; *p* = 0.011). 1,657 participants (57% of the cohort) completed the CANTAB subtests. No significant group differences were found on the PAL or SWM tasks (see supplementary table).

### Olfactory function

**Fig. 7:**
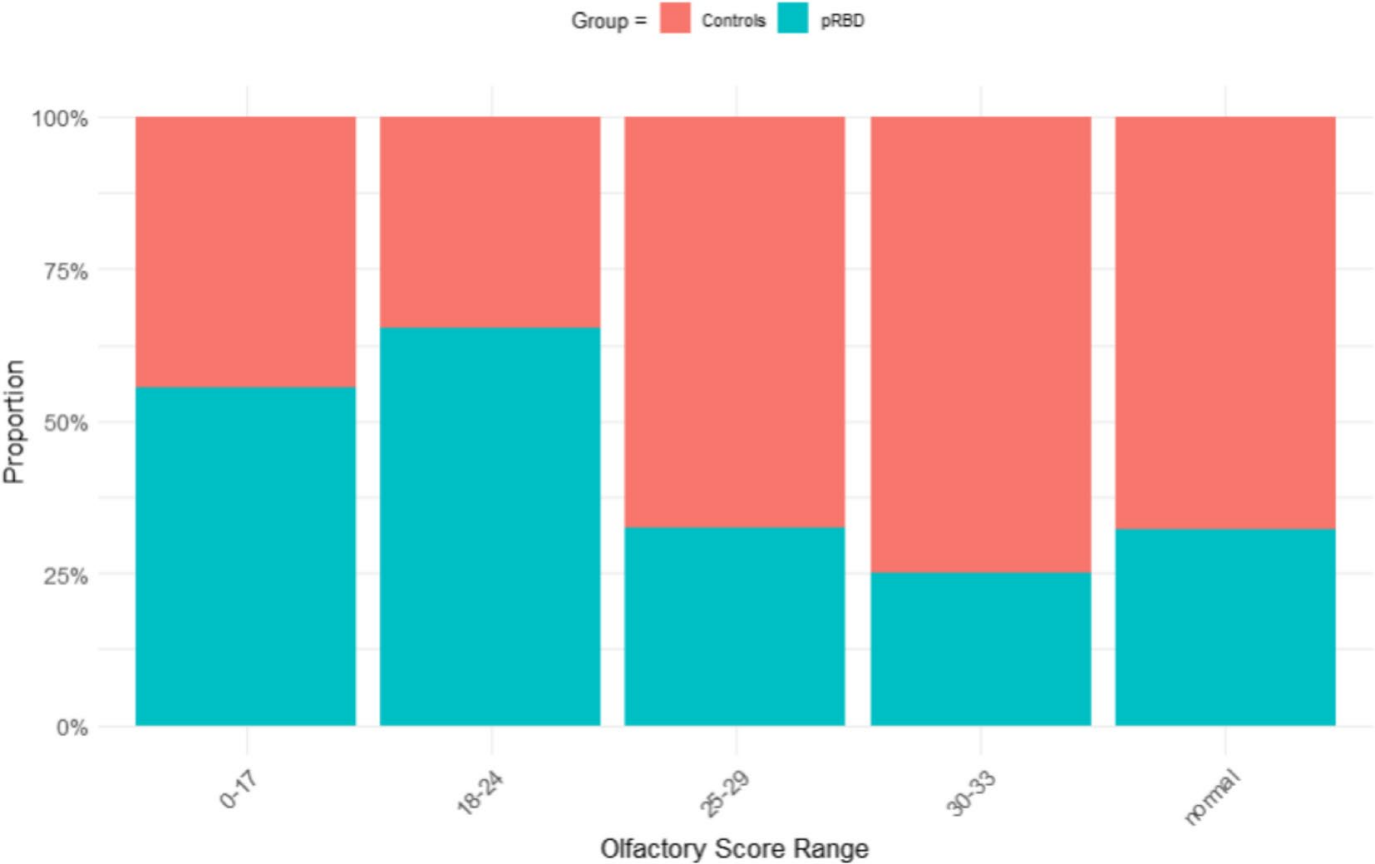
pRBD and control groups stratified by olfactory score range.

304 participants (11% of the cohort) completed UPSIT kits: 121 UK kits and 183 USA versions, with kit-subtype adjusted for in the analyses. Data was extracted from both UK and USA versions, with 33 out of 40 scents identical in each version [55]. Overall, total olfactory scores were found to be lower for the pRBD group (*F* = 6.8730; *p* = 0.01) compared to controls, after adjusting for confounders of age, sex, indigenous origin and kit type (see Table 6). When stratified by olfactory score range, those with pRBD had a greater proportion of olfactory scores in the lower ranges compared to controls (see Fig. 7).

**Table 6:** Mean olfactory scores between pRBD and control groups, with adjusted significance level

	Control (*N* = 217)	pRBD (*N* = 126)	Adjusted *P*-value
**Olfactory Score**			
Mean (SD)	30.2 (4.09)	28.7 (5.85)	0.014
Median [Min, Max]	31.0 [13.0, 37.0]	30.0 [8.00, 38.0]	

## Discussion

This study presents several key findings regarding the early detection of features relevant to α-synucleinopathies in community-dwelling adults. We used unsupervised home-based tools in a large cohort of older adults and found that motor and non-motor features were more common in those with pRBD compared to controls. Specifically, tremor, altered speech, gait disturbance, falls, anxiety, depression, daytime sleepiness, autonomic dysfunction and pain symptoms were reported more often in the pRBD group. Objective measures of both olfactory and hand motor function detected more impairment in those with pRBD. These findings support our hypothesis that α-synuclein-related symptoms and signs will be more frequent in pRBD than healthy controls, and that remote, unsupervised tests are capable of detecting these differences.

In terms of motor symptoms, people with pRBD reported slurred or softer speech and more shaking in their lips, hands, arms and/or legs compared to controls; they also reported feelings of their feet getting stuck, trouble with balance and most notably, more falls than controls. Although none are indicative of a specific α-synucleinopathy [10], there are some trends with, for example, impaired speech and tremor as typical early motor manifestations in people with PD [56], and early falls more common in those with MSA [57]. Generally, neurodegenerative processes start 10 years or more before these motor changes are evident on clinical assessments [58, 59]. However, we found slight differences in hand motor performances on the TAS Test version of the Alternate Tap Test between those with pRBD versus controls, which has also been found in several other iRBD cohorts [60–62]. Most recently, Simonet and colleagues found that in-person, supervised alternate key tap test performance in 33 people with PSG-confirmed iRBD was slower and less rhythmic, especially with dual motor-cognitive tasks, than in 29 age- and sex-matched controls [60]. In addition, these objective motor markers had greater sensitivity and specificity than the clinical rating scale, MDS-UPDRS-3 [60]. Taken together with our wider results, these findings suggest that subtle subjective and objective motor changes are evident in pRBD. By using TAS Test, which is an online unsupervised alternate tapping test that has been shown to be usable and acceptable across a range of ages and levels of computer literacy and education [63], we have a new home-based monitoring tool to assess hand motor function changes easily, remotely, and frequently in this prodromal stage, and thus improve our understanding of the trajectory of iRBD to clinically-manifest α-synucleinopathies.

Hyposmia is one of the most robust predictors of progression from iRBD to an α-synucleinopathy and this is thought to reflect early pathology in the olfactory bulb [64, 65]. Here we found that olfactory scores were 1.8 points lower overall in the pRBD group (mean 28.7 out of 40) compared to controls (mean 30.2), where normal olfactory function in older adults is usually defined as ≥ 34 out of 40 for males and ≥ 35 for females [11, 66], but tends to be lower with advancing age [67]. It was also found that a greater percentage of people with pRBD had scores ≤ 24, indicating severe microsmia or total anosmia [68]. This is noteworthy as several studies have shown that those with iRBD *and* olfactory loss are at highest risk of conversion, specifically to DLB or PD [10, 64, 69–71]. For example, the International RBD Study Group (IRBDSG) multicentre study of 1,280 participants with iRBD, found that over 80% of those with abnormal olfaction went on to develop DLB or PD within 12 years [10, 64]. Thus, the combined use of olfactory testing, with online hand-movement assessment tools may facilitate further condition-specific risk stratification in the ISLAND Sleep Study and other community cohorts.

In contrast to the high rates of hyposmia, no group differences were found on any of the CANTAB cognitive tests, but a small difference in persistent cognitive symptoms was self-reported. This is interesting, as the IRBDSG study found that cognition was the only variable that differed between those who developed DLB and those who developed PD, when measured by brief cognitive tests (Montreal Cognitive Assessment; MoCA) as well as neuropsychological assessments [10]. Given that CANTAB has been validated for detecting early progression of Alzheimer’s disease and PD [45], it may be that the PAL and SWM tasks used in this study are not as sensitive to prodromal changes found in DLB. An alternative explanation is that subtle declines in PAL and SWM are also occurring in controls as part of cognitive age-related decline or preclinical Alzheimer’s disease, causing the effect size between two groups to be less apparent [46]. In addition, the subjective question in this study was administered several months after the CANTAB, therefore some cognitive changes may have been noticed since completion of the objective assessment. Future investigations in the Tasmanian ISLAND Sleep Study will address this issue by inviting participants to attend an interdisciplinary cognitive assessments clinic [72], and administering the MoCA to both people with pRBD and PSG-confirmed iRBD, to clarify whether objective cognitive changes are evident in this cohort, as well as a full neuropsychological battery to those with confirmed iRBD.

Other non-motor features relevant to early α-synuclein-related conditions include constipation, urinary dysfunction, sleep disturbances, pain, and mood disturbance [73–75]. We assessed all 5 of these domains remotely using validated screening tools in an easily accessible online format and completion rates were high for all questionnaires.

Firstly, we found that people with pRBD reported more autonomic features in terms of orthostatic intolerance, vasomotor dysfunction, gastrointestinal dysfunction, and secretomotor dysfunction. Orthostatic intolerance, as gauged by questions asking about feeling faint, dizzy, “goofy”, or experiencing difficulty thinking soon after standing up from a sitting or lying position, is a key prodromal feature of α-synucleinopathy [76, 77]. In a prospective study of 91 people with iRBD, orthostatic intolerance was found to occur up to 20 years before an overt diagnosis of DLB or PD and could identify α-synucleinopathy with high sensitivity (50–90%) up to 5 years before a diagnosis [77].

Gastrointestinal dysfunction (as gauged through questions about constipation, diarrhoea, bloating and abdominal pain), secretomotor dysfunction, such as dry eyes, dry mouth, difficulty swallowing and dry skin, and vasomotor dysfunction (including colour changes in the skin, such as red, white, or purple, and particularly in the hands and feet) were also more common in the pRBD group. These results align with previous reports [78–87], for example research by McCarter and colleagues established that severity of autonomic dysfunction is a prodromal symptom of DLB, more so than PD. In their study of 18 patients with iRBD, 12 went on to develop DLB and PD, and those who developed DLB showed greater autonomic severity scores than those who developed PD [88]. As such, these changes are thought to be related to α-synuclein pathology in the enteric nervous system as well as in the skin or submandibular ganglions decades before phenoconversion [88, 89]. These findings indicate that specific autonomic tests may be key to determining which α-synucleinopathy people with iRBD may progress to, and the fact that the COMPASS-31 questionnaire has been validated against objective measures makes it suitable for remote administration. This is also one of the first studies to examine autonomic dysfunction remotely in a large community cohort using the COMPASS-31, showing that signs and symptoms in those at-risk of α-synucleinopathy can be detected and monitored using this method.

Another key finding was the high prevalence and intensity of pain in the pRBD group compared to controls. Previous research has shown that people with PD report altered sensory and pain thresholds, which is thought to be caused by degeneration of pain processing neurons and pathways in the central nervous system [90, 91] and there is evidence that pain begins at least a decade before a diagnosis of PD as well as throughout the clinical course of PD, DLB and MSA [92]. However, there is very little research into pain and iRBD. The first identified was a 2024 study in 159 war veterans with pRBD (and 67 polysomnography-confirmed iRBD cases), which reported significantly greater chronic pain in veterans with RBD compared to controls [93]. Here we build upon these findings in a larger sample of 235 people with pRBD, finding significantly higher reports of neuropathic and intermittent pain. This is the largest study to date assessing pain in pRBD and the results, taken together with emerging evidence in prodromal PD [90, 94, 95], highlight the importance of recognising pain as a key feature of α-synucleinopathies.

In terms of sleep, the pRBD group reported worse sleep quality, specifically increased sleep disturbance and daytime dysfunction due to sleepiness. Since iRBD involves dream enactment overnight, sleep disturbance is a common theme due to the disruption that motor events have on normal sleep patterns [96]. But other sleep disorders, such as restless leg syndrome, and the circadian rhythm changes associated with DLB, PD, or MSA could also be relevant [97].

As expected, levels of anxiety and depression were substantially higher in people with pRBD; this aligns with previous studies that found mood changes are common α-synuclein-related features and tend to be mild early on, but as the disease progresses, can cause an increased burden and reduced quality of life for people with DLB, PD and MSA [74, 98–100]. It may be that targeting mood disturbance symptoms in the early period of neurodegeneration when future patients are still free of any symptoms, could have a greater impact on the trajectory of disease progression [101].

An incidental, but noteworthy finding from this study is the significant proportion of Aboriginal and/or Torres Strait Islander people who screened positive for pRBD; these people are indigenous to Australia and make up approximately 3.8% of the population [102]. Only 1.3% of the ISLAND Sleep Study cohort identified as Aboriginal and/or Torres Strait Islander, yet 4.3% of these participants made up the pRBD group compared to 0.9% of the control group. To the best of our knowledge, this is the first study to identify an association between indigenous Australian origin and iRBD, and little research on α-synucleinopathy risk has been undertaken in indigenous Australasian populations before [103]. Aboriginal and Torres Strait Islander people are known to experience more adverse health burdens than the rest of the Australian population, with epidemiological studies showing they have a greater prevalence of high blood pressure, heart disease, lung cancer, and diabetes, along with more physical disabilities, alcohol abuse and psychological distress [104, 105]. Alzheimer’s disease prevalence in Aboriginal and Torres Strait Islander people is also three to five times higher than the general Australian population [106], with a possible elevation in *APOE* ε4 allele frequency [107]. These statistics suggest that this population may be at higher risk of neurodegenerative disease more broadly, but further research is needed to explore the potential prevalence of iRBD and α-synucleinopathy risk in the Indigenous Australian population. The ISLAND Sleep Study provides a unique opportunity to do so as we have a record of strong completion rates over time (42% over 4 years) within the broader ISLAND Project [108].

There are several strengths and limitations to this study. The strengths are the inclusion of a large cohort of community-dwelling older adults, living across rural and urban areas with a broad spread of ages, from 50 to 90 years, and a near even sex-split in the pRBD group. We also carefully collected a wealth of additional data and variables to fully understand potential confounding effects in this cohort, and thus adjusted for the significant factors of age, sex and Aboriginal/Torres Strait Islander origin.

We acknowledge that the original sample of the ISLAND Sleep Study is not representative of the general population: it was female predominant and highly educated, and prone to self-selection ‘healthy-bias’, as participants were mainly those interested in dementia prevention with access to a computer. Tasmania itself is not representative of the broader cultural diversity in Australia either, with the majority of its population coming from Northern European ancestries. Although high completion rates were obtained from this cohort, it is not yet known whether attrition will become evident over time. Usability data was not collected, therefore future projects will include questions to elucidate whether the methods used are acceptable to participants. Only one objective test of motor function was used in this project, and plans are underway to broaden future motor assessment batteries with a wider range of hand motor assessments [109], remote gait analyses [110], actigraphy monitoring, and an online test of speech function [111]. Furthermore, the reliance on a screening questionnaire for the pRBD group is also a limitation to this study with the potential for some participants to have screened false-positive on the RBD1Q, especially if they do not have a bed partner. This study is ongoing and home-based vPSGs are currently underway to confirm the presence of iRBD in this population, which will then allow for more specified analyses such as principal component analysis and machine learning to investigate patterns of features that group together within individuals, which will assist in better prediction of phenoconversion to PD, DLB or MSA.

This study found that low cost, remote, self-administered tests result in high quality data for symptoms and signs relevant to α-synuclein-related conditions, and high completion rates with over 49% of participants completing all online assessments. By using this method, we have the ability to track people with pRBD over time in the community, regardless of location, mobility, funding or other factors that can impede access to in-person clinical assessments. Online self-report questionnaires and tests of motor function, combined with validated postal assessments of olfaction, can guide us towards a better understanding of the prodromal stage of α-synucleinopathy and its trajectory towards overt clinical diagnosis. With the advancement of biological determinates of PD/α-synucleinopathy [112–114], this assessment method would also allow for improved, long term follow up of people found to exhibit potential biomarkers, which would greatly inform the predictability of these new prodromal tests.

Taken together, the findings of this study supplement the evidence that symptoms and signs relevant to α-synuclein-related conditions begin in the prodromal stages alongside pRBD many years before overt phenoconversion. A combined set of easily accessible, low-cost online tools and remote olfactory testing may aid researchers and clinicians in identifying people in the community who are at high risk for developing an α-synucleinopathy. Early detection of risk in the community will improve recruitment into clinical trials, longitudinal tracking and a deeper understanding of potentially disease modifying interventions into the future.
